# Dietary Intake Pattern and Nutritional Status of Women of Reproductive Age in Slum Areas of Pokhara Metropolitan

**DOI:** 10.1155/2024/6677529

**Published:** 2024-09-06

**Authors:** Babita Kandel, Dhurba Khatri, Arun Kumar Koirala, Yamuna Chhetri, Alisha Manandhar

**Affiliations:** ^1^ School of Health and Allied Sciences Pokhara University, Kaski, Pokhara, Nepal; ^2^ Kathmandu Institute of Child Health, Hepaliheight, Kathmandu, Nepal; ^3^ Patan Academy of Health Sciences, Lagankhel, Lalitpur, Nepal

## Abstract

**Introduction:**

The pattern of diet intake is associated with food availability and the intake of nutrients from different food groups and is an important component of nutritional status. The dietary habits of individuals are integral to understanding their nutritional wellbeing, making this assessment crucial for public health interventions. The objective of the study was to assess the dietary intake pattern and nutritional status among women of reproductive age in slum areas of Pokhara Metropolitan. *Methodology*. A cross-sectional analytical study was conducted to assess the dietary intake pattern and nutritional status among 404 women of reproductive age in the slum area of Pokhara from June 2019 to December 2019. A simple random sampling technique was used to select the wards while a purposive sampling technique was used to collect the samples. 24-hour recall meal-based questions and anthropometric measurements were used for collecting data. Data were entered into EpiData and analyzed using SPSS version 16 software for analysis. Frequency and mean, median, chi-square test, and logistic regression were performed.

**Results:**

The mean (±SD) age (SD) of the participants was 31 (±9.25) years, and most of the participants belonged to the age group 25–35 years. Out of 404 women of reproductive age, 99% of the participants consumed starchy staple foods daily, while 56.7% ate vegetables daily, and nearly half frequently consumed prepackaged foods and soft drinks. Among the total participants, 41.3% were found with a normal BMI, 37.6% were overweight, and only 12.4% were underweight. There was a significant association between underweight and age, women's occupation, and frequency of food consumption per day. Age, knowledge of nutrition, and frequency of food consumption per day were found to be associated factors with being overweight at a *p* value <0.05.

**Conclusion:**

Age, occupation status, and frequency of food consumption were the key predictors of being underweight, and age, knowledge regarding nutrition, and frequency of meals were the key predictors of being overweight. Developing countries must emphasize the importance of considering these factors in public health interventions and strategies aimed at promoting healthy weight management. More studies are needed.

## 1. Introduction

The dietary intake pattern means the consumption of food items or food groups over a period of time [1]. It is an essential element of diet quality. Consuming a variety of foods across and within food groups is associated with a sufficient intake of essential nutrients and promotes good health [2, 3]. The nutritional status is an indication of the general goodness of a population [4]. Normal nutritional status is managed by balanced food consumption and normal use of nutrients. This means consumption of vitamins and minerals in an inadequate pattern leads to low nutritional status [3].

The dietary consumption pattern plays a vital role in human health. Mostly in low-income countries and low-income settings, people consume inadequate and insufficient nutrients due to resource limitations [5]. Especially women of reproductive age (15–49 years) are nutritionally vulnerable due to the physiological requirements of pregnancy and lactation. Nutrition requirement is also higher in nonpregnant and nonlactating women [6]. Studies show that there is good nutritional status in pregnant and lactating women compared to other women of reproductive age due to adequate dietary intake [7]. People living in backward and drought-prone rural areas and urban slums are highly backward in the consumption of an adequate diet due to unavailability and lack of access to a variety of foods [8]. Insufficient dietary intake, micronutrient deficiencies, and infection may result in malnutrition [9]. Poor nutrition in women of reproductive age, exacerbated by challenges in accessing an adequate diet in slum areas, poses significant health implications. Many women suffer from a combination of chronic disorders, underweight and anemia, and many micronutrient deficiencies including infections such as HIV and malaria [10]. Stunting and low body mass index among mothers increase the risk of fetal growth restriction, obstructed labor, and maternal and neonatal deaths [9].

Rural and urban slum areas often face food insecurity and limited food variety. In Nepal, particularly among women of reproductive age, improper and insufficient dietary consumption patterns have led to a deficiency of essential nutrients, especially during pregnancy and lactation [11], where 41% of women of reproductive age are anemic, which has increased from 35% in 2011 to 41% in 2016 [12].

Different factors are associated with the nutritional status among women of reproductive age, such as sociodemographic, socioeconomic, behavioral, nonbehavioral, and different community factors. Socioeconomic and behavioral factors are mainly associated with the nutritional status and dietary intake patterns [10]. Other factors include inadequate care practices and poor status of women, low quality and accessibility of healthcare services, food insecurity, low food access, and unhygienic household environments [13]. This multifaceted vulnerability has the potential to give rise to diverse health issues and escalated pregnancy risks, underscoring the urgent imperative to promptly address nutritional disparities within this population [14]. Studies on the nutritional status of women in Nepal have primarily focused on rural areas, with limited data available on the nutritional status and dietary intake patterns of women in urban slums. The objective of the study was to assess the dietary intake pattern and nutritional status among reproductive-age women in slum areas of Pokhara metropolitan.

## 2. Materials and Methods

### 2.1. Study Design and Setting

A community-based cross-sectional study was conducted in the slum area of Pokhara metropolitan city, Gandaki Province, Nepal, from June 2019 to December 2019. As per the Pokhara Metropolitan Office, there were approximately 3,746 households situated in areas characterized by limited space, overcrowding, and inadequate housing conditions.

### 2.2. Study Population and Study Unit

In this study, all women of reproductive age (15–49 years) in the slum area of the Pokhara metropolitan were taken as the study population and women of reproductive age (15–49 years) in this slum area were taken as the study unit. Lactating and pregnant mothers, physically sick (chronic diseases), mentally retarded, and handicapped women of reproductive age were excluded.

### 2.3. Sample Size

Sample size was calculated with the expected prevalence of 50%, 5% margin of error, and alpha 0.05. The sample size thus obtained was 384 using the Cochrane formula, i.e., *n* = *Z*^2^*pq*/*d*^2^. Adding 5% of the nonresponse rate to it, the final sample size hence obtained was 404.

### 2.4. Sampling Methods

Out of 33 wards, only 10 wards containing slum areas in Pokhara [15], and five wards were selected using a simple random technique using the ballot method. Subsequently, within the selected wards, a purposive sampling technique was employed to purposely identify and choose to represent the target population of reproductive-age women in slum areas, enhancing the relevance and focus of the research.

### 2.5. Data Collection Tools and Technique

The data were obtained through face-to-face interviews using a quantitative approach. A semistructured questionnaire was used as the data collection tool. Measuring instruments such as a weight bathroom scale, a weighing machine, and a height measurement tape for height measurement were used. Semistructured questionnaire includes sociodemographics, food frequency measures, and 24-hour recall.

We have adopted and modified the nutritional knowledge questionnaires from the Adolescent Nutrition Survey in Nepal, 2014 [16]. For body measurements, we recorded height and weight. Dietary intake patterns were assessed using a questionnaire for dietary diversity, a questionnaire adapted from the Food and Agricultural Organisation (FAO) guidelines [17].

The open recall method involved continuous probing to ensure complete recall of the food consumed. In this method, the participants responded about the food item they consumed or did not consume, respectively, during the past 24 hours. The nutrition status was assessed by measuring the weights of women with a battery-powered digital scale and their heights to the nearest centimeter using a height scale following standard anthropometric techniques. During weight and height measurements, respondents removed their shoes and jackets and were asked to wear light clothing.

### 2.6. Study Variable

#### 2.6.1. Dependent Variable

The dependent variable was the nutritional status of women of reproductive age, i.e., underweight and overweight.

#### 2.6.2. Independent Variables

Attributes such as age, type of family, religion, education level, marital status, women's occupation status, monthly income, weekly food consumption pattern, height, and weight were the independent variables.

### 2.7. Operational Definition

Dietary intake pattern: amount of foods such as starchy staples, fruits, vegetables, meat and fish, egg, legumes, milk, and milk products consumed in the last seven days by women of reproductive age.

Nutritional status: the nutritional status of women of reproductive age was measured in terms of the body mass index (BMI) value and the BMI value was categorized as per the World Health Organization [18].

Underweight: women of reproductive age whose BMI was less than 18.5 were classified as underweight. It was dichotomized as underweight (if BMI <18.5) and not underweight (if BMI ≥25)

Overweight: women of reproductive age whose BMI was more than or equal to 18.5 were classified as underweight. It was dichotomized as overweight (if BMI ≥25) and not overweight (if BMI <25).

### 2.8. Data Processing and Analysis

The data collected were cleaned and edited on the day of data collection, and EPI-DATA version 3.1 was used for data entry and checks were defined. Then, the entered data were exported to Statistical Package for the Social Sciences (SPSS) version 16.0 for further analysis. The analysis was done after developing a data analysis plan. The body mass index (BMI) was calculated by dividing the weight in kilograms by the height in meters squared (kg/m^2^), providing a numerical value used to categorize individuals as underweight, normal weight, overweight, or obese. The dietary intake data were analyzed by categorizing the frequency of food item consumption over the past week into four groups as follows: never, sometimes (1–3 times per week), often (4–6 times per week), and daily. The data were summarized in terms of frequency and mean or median based on the normality of the data (by applying the Kolmogorov–Smirnov test). A chi-square test and logistic regression were performed to find an association between dependent and independent variables, taking a 95% confidence interval.

### 2.9. Data Quality Control

Hard copies of the collected data were safely stored in distinct record files, each assigned a specific code. Pretesting was done in a similar 10% of the sample size and adjustments were made based on the results. Data preservation was continued after entry. Check recheck, double entry, and data clearance were done for the quality assurance of the data.

### 2.10. Ethical Considerations

Ethical approval was taken from the Institutional Review Committee (IRC), Pokhara University (ref. no. 104/076/077). Before data collection, written informed consent was obtained from each participant and the purpose and objectives of the research were discussed. In order to maintain confidentiality, the personal names of the participants were omitted, and a unique identity number system was used.

## 3. Results

### 3.1. Sociodemographic Characteristics of Participants

The mean age of the respondents was 31.84 with S.D. 9.251 years. Most of the participants were of the nuclear family (74%) and were found to follow the Hindu religion (75%). Most of the participants had basic education (46.5%) and 7.2% were illiterate. Almost three quarters of the participants were found to be married. In total, 32.7% were involved in daily wage labor (32.7%), followed by the housewife. A majority (63.9%) of them had an income in the range 13500–25000 (Table 1).

### 3.2. Weekly Food Consumption by Participants

Almost 99% of the participants had starchy staple foods every day of the week, while 44.8% of those polled ate legumes, nuts, and seeds 1–3 times. Participants who never consumed fruits in a week were found to be 7.7%, while 56.7% consumed vegetables daily. Meat and fish were consumed 1–3 times a week by 45.5% and 76.7%, respectively, and 70.3% consumed eggs on time in a week mostly. Similarly, 39.9% of the participants never consumed milk or dairy products. Almost half (49%) of the participants had consumed prepackaged foods frequently, 48% had soft drinks 1–3 times a week, and most participants had consumed snacks such as samosa and momo. It shows that almost half (48%) of the respondents consumed soft drinks 1–3 times a week, 49% consumed 4–6 packaged foods, and more than half (68.6%) consumed 1 junk food 1–3 times a week (Table 2).

### 3.3. Anthropometric Measurement

The majority of the participants were within a normal BMI range, that is, 41.3%, 37.6% of the participants were underweight, 12.4% were overweight, and 8.7% of the participants were found obese with 154.15 ± 4.8 centimeters (cm) and 56.93 ± 9.397 kilogram (kg) mean ± S.D. of height and weight, respectively (Table 3)

### 3.4. Association between Underweight and Sociodemographic Factors

In the multivariate binary logistic regression model, age, occupation of women, and frequency of food consumption were significantly associated with being underweight at the *p* value <0.05. The respondents <30 years were about 9 times more likely to be underweight than those <30 years (AOR: 9.41 and 95% CI: 1.86–47.59). Similarly, unemployed women were about 3 folds more likely to be underweight than employed women (OR: 3.83 and 95% CI: 1.712–8.596), taking into consideration women's occupations. (Table 4). ^∗^Significant at *p* value <0.05, ^∗∗^significant at *p* value <0.001.

### 3.5. Association between Overweight and Sociodemographic Factors

Age, knowledge of nutrition, and time of food consumption per day were significantly associated with being overweight at the *p* value <0.05. The respondents >30 years were 6 folds more likely to be overweight than <30 years (OR: 6.34 and 95% CI: 3.58–11.22). Similarly, those women who didn't know about nutrition were about 2 folds likely to be overweight than those who knew (OR: 2.08 and 95% CI: 1.22–3.51) (Table 5).

## 4. Discussion

This study shows that nearly half (41.3%) of the population had a normal BMI (18.5–24.9), 37.6% were overweight, 12.4% were underweight, and the least of the respondents (8.7%) were obese. According to NDHS 2016, 17% were underweight, 17% were overweight, and 5% were obese [12]. Another study conducted in Nepal showed that 32.3% were underweight and 48% were overweight or obese [10].

The present study among women of reproductive age revealed that nearly half of the respondents consumed 3 times food per day, which is similar to the study conducted in North India [19]. Almost all respondents take starchy staple food daily, which is in agreement with the findings of a study conducted in Nepal [11] and greater with a study conducted in Punjab, India [3]. This study showed that 3/4 of the respondents take 1–3 times a week but the study conducted in Punjab showed that only 14.8% of the respondents take fruits 1–3 times a week [3]. According to this study, more than half of the participants took green vegetables daily for a week, which is supported by the findings from a study in Lahore, Pakistan [20]. This study found that 39.9% of the participants never consumed milk or milk products, while only 1.3% of the participants never consumed milk and milk products in the rural community of Lahore, which was less than in the present study [20]. Considering these dietary patterns, women in slum areas may be vulnerable to nutritional imbalances. However, starchy staples are eaten daily, and infrequent vegetables, fruits, legumes, and milk raise concerns about micronutrient deficiencies. Moreover, the significant proportion of consumption of soft drinks, packaged foods, and junk food may contribute to poor nutritional status for women and increase health issues. This finding was also supported by the different studies [11, 21].

The present study revealed that a variety of factors are associated with the nutritional status of women of reproductive age. Age was significantly associated with being underweight, which states that individuals <30 years of age are comparatively found to be underweight than those <30 years of age. This finding is similar to the study conducted in Nepal [22] and in our neighboring countries, India [4, 23, 24], and Bangladesh [25] showed that a greater percentage of underweight women were in the lower age groups, whereas those who were overweight and obese were in the higher age groups. This finding might be because women 30 years of age are usually pregnant and lactating women who are bound to focus more on nutritious food intake according to the societal norms of the Nepalese context, resulting in more weight gain than those under 30 years of age. Contrary to the abovementioned findings, a community-based cross-sectional analytical study conducted in Lahan municipality of Siraha district of Nepal from June to December 2017, revealed that nutritional status decreases with increasing age [26].

This study showed that *w* unemployed women were at an increased risk of being overweight, which was supported by a study by Bhandari et al. [11]. It could be the impact on BMI supported by evidence showing different effects of stress and financial restrictions on energy balance, including individual variations in stress, eating, and food choices on a restricted budget [27–29].

Similarly, being underweight was significantly associated with times of daily food consumption, with less time of food consumption per day being more prone to obesity, similar to results reported in Nepalese villages [11]. It could be due to infrequent meals, skipped meals or prolonged fasting, limited availability of food throughout the day, and contributes to lower calorie intake.

Similarly, if we look at the association between overweight and factors, a significant statistical association was found with age. Increased age was more likely to be overweight. This finding is consistent with the results of the study conducted in India [23], Bangladesh [30], Ethiopia [31], and Maldives [32] among women whose increasing age contributed to obesity. This is likely due to the evidence that changes in body fat distribution and metabolism with aging may ultimately lead to a vicious cycle that accelerates aging and causes age-related diseases [33]. In contradiction to this, a study conducted in Malawi showed a decrease in overweight patterns with increasing age [34].

Similarly, statistical significance was found between obesity and knowledge about nutrition and the frequency of eating meals. Those women who had nutrition-related knowledge were less likely to be overweight. This finding was supported by the study by Shrestha et al. [35] and Akkartalaand and Gezer [36]. However, this finding is contrary to the findings reported in studies conducted in similar settings, indicating that women with formal education were more likely to be malnourished [11, 37]. The variance in this finding may be due to differences in formal education, carelessness about body weight, and lack of adequate knowledge of healthy food choices.

In addition, women who ate four or more than four meals per meal were more likely to be overweight. It means that increased meal frequency was associated with greater weight and was supported by different studies [38–40]; however, some studies claim that the body mass index decreased with increasing meal frequency [41, 42].

The limitation of this study is that the quantity of food items was not included.

## 5. Conclusions

A significant proportion of women were underweight and overweight. A woman's age, occupation, and frequency of food consumption were significant predictors of being underweight, while an individual's age, knowledge of nutrition, and frequency of food consumption per day were significant predictors of being overweight. A complex interplay among demographic, lifestyle, and dietary factors is revealed by these findings. Developing countries must prioritize the incorporation of key predictor factors in public health interventions and strategies to promote healthy weight management. This emphasis should encompass targeted initiatives, such as nutritional education and the promotion of balanced diets, contributing to the enhancement of the overall health of women of reproductive age. In addition, longitudinal and intervention studies must be conducted.

## Figures and Tables

**Table 1:** Sociodemographic characteristics of participants.

Variables		Frequency (*n*)	Percent (%)
Age groups	15–25	113	28
	25–35	143	35.4
	35–45	116	28.7
	45–49	32	7.9
	Mean: 31.84, median: 33, S.D.: 9.251, min.: 15, max: 49		

Type of family	Nuclear	299	74
	Joint	93	23
	Extended	12	3

Religion	Hindu	303	75
	Muslim	66	16.3
	Buddhism	35	8.7

Education level	Basic	188	46.5
	Just literate	100	24.8
	Secondary	86	21.3
	Illiterate	29	7.2
	Bachelors	1	0.2

Marital status	Married	306	75.7
	Unmarried	56	13.9
	Widow	32	7.9
	Divorced	10	2.5

Women's occupation	Daily wage labor	132	32.7
	Housewife	109	27.0
	Business	77	19.1
	Student	36	8.9
	Service: gov./private	29	7.2
	House keeping	19	4.7
	Agriculture	2	0.5

Monthly income	Less than 13500	35	8.7
	13500–25000	258	63.9
	More than 25000	111	27.5
	Mean: 23158.42, median: 20000, min: 5000, max: 60000		

**Table 2:** Weekly food consumption by participants.

Food items	Never	Sometimes (1–3 times)	Often (4–6 times)	Daily
Starchy staples	0	4 (1%)	0	400 (99%)
Vegetables	1 (0.2%)	59 (14.6%)	115 (28.5%)	229 (56.7%)
Legumes, nuts, and seeds	1 (0.2%)	181 (44.8)	138 (34.2)	84 (20.8%)
Milk and milk products	161 (39.9%)	151 (37.4%)	13 (3.2%)	79 (19.6%)
Meat and fish	24 (5.9%)	184 (45.5%)	124 (30.7%)	72 (17.8%)
Fruits	31 (7.7%)	310 (76.7%)	46 (11.4%)	17 (4.2%)
Eggs	65 (16.1%)	284 (70.3%)	43 (10.6%)	12 (3%)
Packaged foods	26 (6.4%)	153 (37.9%)	198 (49)	27 (6.7%)
Soft drinks	49 (12.1%)	194 (48%)	152 (37.6%)	9 (22%)
Snacks food	21 (5.2%)	277 (68.6%)	99 (24.5%)	7 (1.7%)

**Table 3:** Anthropometric measurement.

Variable	Frequency	Percent (%)
*Height*		
Less than or equal to 145 cm	14	3.5
More than 145 cm	390	96.5
Mean ± S.D., min, max	154.15 ± 4.8, 142.24, 165.15	

*Weight*		
Less less than or equal to 45 kg	67	16.6
More than 45 kg	337	83.4
Mean ± S.D., min, max	56.93 ± 9.397, 34,79	

*BMI*		
Normal range (18.5–24.99)	167	41.3
Overweight (25–29.9)	152	37.6
Underweight (<18.5)	50	12.4
Obese (≥30)	35	8.7
Mean ± S.D., min, max	24.00 ± 4.15, 15.32, 33.64	

**Table 4:** Association between underweight and sociodemographic factors.

Variables	Underweight				Unadjusted OR (CI at 95%)	*P* value	Adjusted OR (CI at 95%)	*P* value
	Yes *n* (%)		No *n* (%)					
*Age*								
<30	48	31.2	106	68.8	13.811 (3.24–58.82)	<0.001^∗∗^	9.41 (1.86–47.59)	0.007
≥30	2	3.2	61	61	Ref.		Ref	

*Marital status*								
Married	21	13.8	131	86.2	0.199 (0.102–0.390)	<0.001^∗∗^	2.264 (0.981–5.225)	0.056
Single	29	44.6	36	55.4	Ref.		Ref	

*Women's occupation*								
Employment	18	12.9	122	87.1	Ref.		Ref	
Unemployment	32	41.6	45	58.4	0.207 (0.106–0.406)	<0.001^∗∗^	3.83 (1.712–8.596)	0.001

*Knowledge about nutrition*								
Yes	46	26.9	125	73.1	Ref.		Ref	
No	4	8.7	42	91.3	3.864 (1.31–11.37)	0.009^∗∗^	1.35 (0.373–4.708)	0.664

*Snacks food*								
Sometime	24	17.6	112	82.4	0.453 (0.239–0.86)	0.014^∗^	(0.463–2.110)	0.989
Daily	26	32.1	55	67.9	Ref		Ref	

*Times of food consumption per day*								
2	4	40	6	60.0	0.212 (0.053–0.84)	0.006	1.870 (0.35–9.88)	0.461
3	32	34.0	62	66.0	0.274 (0.136−0.0.55)	0.629	6.85 (1.29–36.28)	0.024
4 or more than 4	14	12.4	99	87.6	Ref	<0.001^∗∗^	Ref	

^∗^Significant at *P* value <0.05, ^∗∗^significant at *P* value <0.001.

**Table 5:** Association between overweight and sociodemographic factor**s**.

Variables	Overweight				UOR (CI at 95%)	*P* value	AOR (CI at 95%)	*P* value
	Yes		No					
*Age*								
<30	30	22.1	106	77.9	Ref	<0.001^∗^	Ref	<0.001^∗∗^
>30	157	72.0	61	28.0	0.110 (0.067–0.182)		6.46 (3.65–11.40)	

*Knowledge about nutrition*								
Yes	83	39.9	125	60.1	Ref	<0.001^∗∗^	Ref	
No	104	71.2	42	28.8	0.268 (0.170–0.422)		2.09 (1.23–3.54)	0.006^∗^

*Smoking*								
Yes	36	67.9	17	32.1	2.104 (1.132–3.908)	0.017	1.48 (0.707–3.131)	0.295
No	151	50.2	150	49.8	Ref			

*Times of food consumption per day*								
2	30	83.3	6	16.7	Ref	<0.001^∗^	Ref	
3	88	58.7	62	41.3	0.139 (0.055–0.353)	<0.001^∗^	1.76 (0.61–4.4.766)	0.308
4 or more than 4	69	41.1	99	58.9	0.491 (0.314–0.768)	0.491	3.34 (1.212–9.213)	0.020^∗^

*Junk food*								
Sometimes	162		112	40.9	Ref	<0.001^∗^	Ref	
Often/daily	25	31.2	55	68.8	3.182 (1.87–5.409)		1.235 (0.651–2.343)	0.517

*Food taboo practice*								
Yes	32	76.2	10	23.8	3.241 (1.54–6.82)	0.001	1.92 (0.814–4.526)	0.131
No	155	49.7	157	50.3	Ref		Ref	

^∗^Significant at *P* value <0.05, ^∗∗^significant at *P* value <0.001.

## Data Availability

The data that support the findings of this study are available from the corresponding author upon reasonable request.
